# Sea Louse Infection of Juvenile Sockeye Salmon in Relation to Marine Salmon Farms on Canada's West Coast

**DOI:** 10.1371/journal.pone.0016851

**Published:** 2011-02-09

**Authors:** Michael H. H. Price, Stan L. Proboszcz, Rick D. Routledge, Allen S. Gottesfeld, Craig Orr, John D. Reynolds

**Affiliations:** 1 Department of Biology, University of Victoria, Victoria, Canada; 2 Raincoast Conservation Foundation, Sidney, Canada; 3 Watershed Watch Salmon Society, Coquitlam, Canada; 4 Department of Statistics and Actuarial Science, Simon Fraser University, Burnaby, Canada; 5 Skeena Fisheries Commission, Hazelton, Canada; 6 Earth to Ocean Research Group, Department of Biology, Simon Fraser University, Burnaby, Canada; University of Western Ontario, Canada

## Abstract

**Background:**

Pathogens are growing threats to wildlife. The rapid growth of marine salmon farms over the past two decades has increased host abundance for pathogenic sea lice in coastal waters, and wild juvenile salmon swimming past farms are frequently infected with lice. Here we report the first investigation of the potential role of salmon farms in transmitting sea lice to juvenile sockeye salmon (*Oncorhynchus nerka*).

**Methodology/Principal Findings:**

We used genetic analyses to determine the origin of sockeye from Canada's two most important salmon rivers, the Fraser and Skeena; Fraser sockeye migrate through a region with salmon farms, and Skeena sockeye do not. We compared lice levels between Fraser and Skeena juvenile sockeye, and within the salmon farm region we compared lice levels on wild fish either before or after migration past farms. We matched the latter data on wild juveniles with sea lice data concurrently gathered on farms. Fraser River sockeye migrating through a region with salmon farms hosted an order of magnitude more sea lice than Skeena River populations, where there are no farms. Lice abundances on juvenile sockeye in the salmon farm region were substantially higher downstream of farms than upstream of farms for the two common species of lice: *Caligus clemensi* and *Lepeophtheirus salmonis*, and changes in their proportions between two years matched changes on the fish farms. Mixed-effects models show that position relative to salmon farms best explained *C*. *clemensi* abundance on sockeye, while migration year combined with position relative to salmon farms and temperature was one of two top models to explain *L*. *salmonis* abundance.

**Conclusions/Significance:**

This is the first study to demonstrate a potential role of salmon farms in sea lice transmission to juvenile sockeye salmon during their critical early marine migration. Moreover, it demonstrates a major migration corridor past farms for sockeye that originated in the Fraser River, a complex of populations that are the subject of conservation concern.

## Introduction

Pathogens are growing threats to wildlife [1], [2]. The spread of infectious pathogens commonly occurs when humans bring wildlife into increased contact with infected domestic animals [3], [4]. Ensuing epizootics have devastated wild populations, as illustrated by the transmission of rabies from domestic dogs to wild carnivores [5], [6], *Pasteurella* from domestic to wild sheep [7], and *Crithidia bombi* from commercial to wild bumble bees [4].

Caligid sea lice (mainly *Lepeophtheirus salmonis* and *Caligus* spp.) are the most widespread marine parasites affecting domestic and wild fish, and have now emerged as important pathogens in many coastal marine areas [8]–[10]. Sea lice feed on surface tissues of their hosts, which can lead to many problems especially for small juvenile fish [8], [11]. Sea lice can compromise osmoregulation [12], induce behavioral changes that increase predation risk [13], reduce growth rates and, in sufficient numbers, result in host death [9], [14], [15]. Sea lice also have been shown to serve as vectors for the spread of fish diseases [16], [17].

The transmission of pathogens to wildlife frequently occurs where host populations are concentrated into dense aggregations [6], [18]. The recent global expansion of marine salmon farming is one such situation in which concentrated reservoir populations may dramatically alter the natural transmission dynamics of salmonid host-parasite systems [9], [19]–[21]. In natural systems, migratory allopatry (the spatial separation of age classes) of wild salmon creates a barrier to parasite transmission [22]. Conversely, salmon farms hold domestic fish, mainly Atlantic salmon (*Salmo salar*), in high densities for months in the same location (i.e., 15–30 kg/m^3^ for up to 24 months) [23]. These crowded conditions facilitate parasite and disease transmission within the farm, and enable exponential population growth of pathogens and release to the surrounding environment [24], [25]. Juvenile wild salmon swimming past salmon farms are frequently infected with sea lice [21], [26], and studies have implicated sea lice from farms in the decline of some wild salmonid populations in Europe and North America [9], [27], [28].

Recent research has raised concern that sea lice from salmon farms may infect juvenile sockeye salmon (*Oncorhynchus nerka*) in an area of Canada's west coast between Vancouver Island and the mainland known as the Discovery Islands [29]. This region is home to the northeast Pacific's largest salmon farm industry and hosts one of the largest migrations of salmon in the world (primarily to and from the Fraser River) [30]. Sockeye is the Pacific Ocean's most economically and culturally important salmon species, and several populations from the Fraser River are endangered [31]. Productivity of Fraser River sockeye has been declining since the early1990s, with 2009 being the lowest on record, prompting the Canadian government to launch a Judicial Inquiry to investigate the cause of the decline and identify imminent threats to their survival [32]. The early marine phase of sockeye remains one of the least understood [33], yet has received the most attention in the search for answers to declining sockeye productivity [34]. Thus, determining whether sockeye are at risk to sea lice transmission from salmon farms during their early marine migration is highly relevant to conservation and management efforts.

In this study we examined parasite infection of wild juvenile sockeye from two geographically separated regions of Pacific Canada: one with salmon farms, and one without. Within the farm region, we compared infection rates on fish from locations that vary in their exposure to farms. We used molecular genetics techniques to determine the origins of the fish, and we employed mixed-effects modelling to examine factors that best explain sea lice abundance.

## Materials and Methods

### Ethics statement

All juvenile salmon were humanely euthanized in accordance with Fisheries and Oceans Canada's national guidelines, under permit XR 21 2007–2008. Study approval by academic ethics committees was not necessary as no academic institution was involved during the data collection.

### Study area and sampling

**Figure 1:**
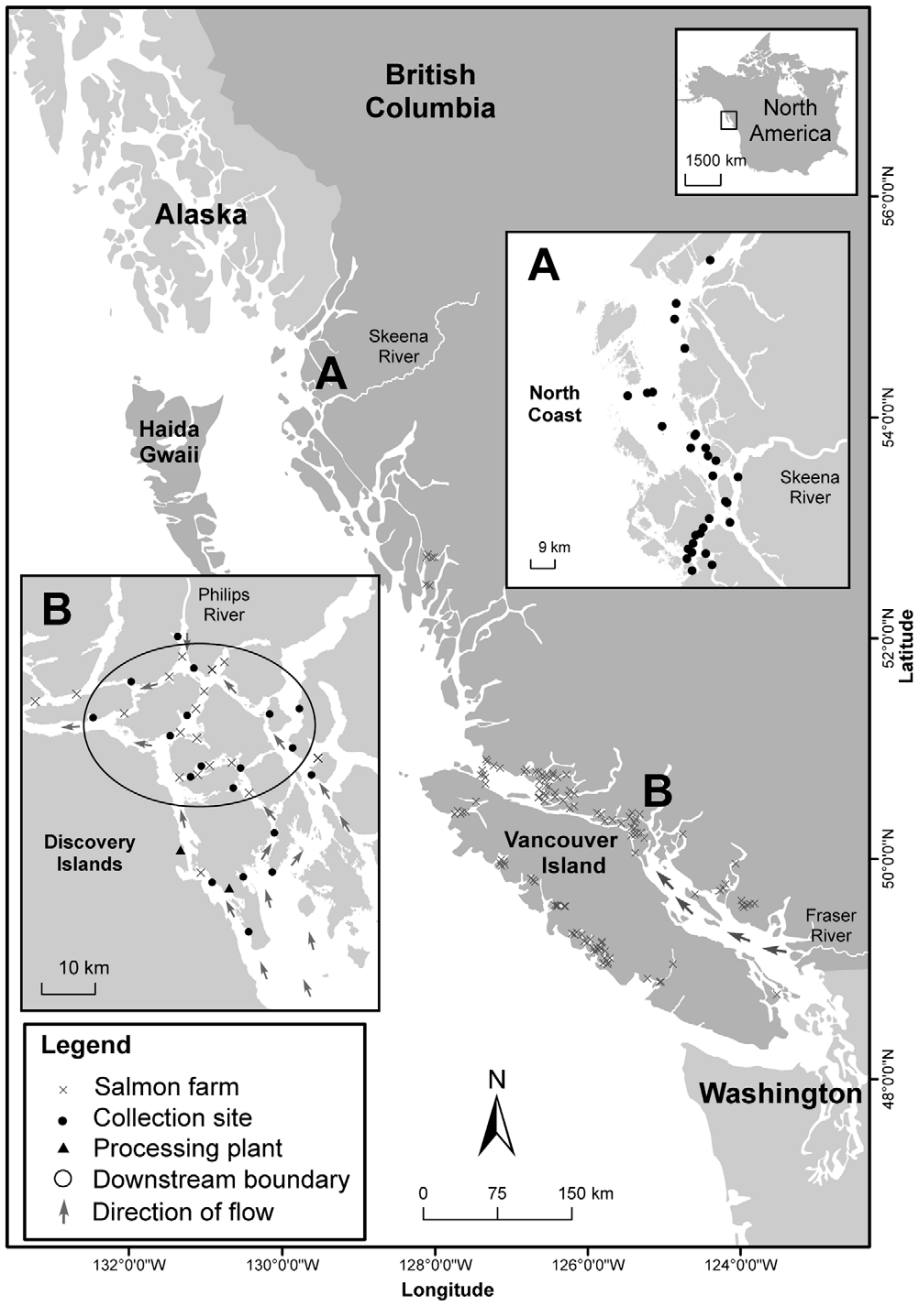
Sockeye salmon collection sites relative to salmon farms. Legend: Downstream boundary encircles all sockeye collection sites situated downstream of at least one salmon farm given the direction of prevailing oceanic flow and migration direction; all other collection sites are considered upstream.

We collected juvenile sockeye from marine waters surrounding the Discovery Islands, an area containing 18 active salmon farms, from April 22 to June 15, 2007 (n = 381) and May 31 to July 3, 2008 (n = 510), and from the north coast of British Columbia, an area without salmon farms, from May 26 to July 5, 2007 (n = 369; Figure 1). Up to five replicate sets of samples were obtained from each site, each year, in the Discovery Islands (1–50 juvenile sockeye salmon per sample), and during 2007 on the north coast (1–129 juvenile sockeye salmon per sample). We used a beach seine (50 m long, 1.5 m deep, 6 mm mesh) among the Discovery Islands to capture sockeye, and a surface trawl-net (18 m long, 5 m opening, 4.6 m deep) on the north coast. The trawl-net was fitted with a rigid holding box at the far end designed for live capture and to minimize the loss of scales and ectoparasites [35]. We recorded sea surface salinity and temperature during each sampling event in both regions using a YSI-30 SCT meter. Fish were immediately frozen and labeled for subsequent laboratory analyses in which individual fish were thawed and assayed for sea lice using a dissecting microscope. Species of motile (i.e., sub-adult and adult) stages of sea lice were directly identified by morphology [36], [37]; younger copepodid and chalimus stage lice were removed from the fish, mounted on permanent slides and examined under a compound microscope for determination based on detailed morphology [36], [37].

### Genetic analyses

We proportionately sampled previously frozen tissues for genetic determination in the Discovery Islands from juveniles retained at each capture location, per sampling event, each year (i.e., 1/3 from 2007, n = 92; 1/5 from 2008, n = 114), and placed them individually in vials of 95% ethanol. We collected fresh tissue from all sockeye (n = 478) on the north coast, and placed them individually in vials of 99% ethanol. Tissue samples from both regions were analyzed at the Fisheries and Oceans Canada (DFO) molecular genetics laboratory in British Columbia. DNA was extracted from tissue [38], and samples were analyzed for polymerase chain reaction products at 14 microsatellite loci [39]. We considered amplification at a minimum of 7 loci as adequate for estimating stock origin as previous surveys of the microsatellite variation in Fraser River sockeye at 6 loci indicated differentiation among populations [38]. Individuals were assigned to source populations using mixed stock analysis techniques employing Bayesian mixture modeling [40] using the software program cBayes. Stock proportions were determined by comparing one mixture (north coast 2007) to a baseline comprising 227 sockeye populations, and two mixtures (Discovery Islands 2007 and 2008) to a baseline comprising 85 sockeye populations [39], [41]. The reported stock composition estimates with corresponding standard deviations were derived from combined posterior distributions using the last 1 000 iterations from 10 Monte Carlo Markov runs of 20 000 iterations.

### Statistical analyses

To test for spatial patterns in sea lice on sockeye, we organized capture locations within the Discovery Islands based on whether each site was: upstream (a position on the juvenile sockeye migration route where fish likely had not passed a salmon farm), or downstream (a position where fish must have passed at least one salmon farm), given the net movement of juvenile sockeye through the region [42]; downstream collection sites are encircled within Figure 1. The ocean environment surrounding the Discovery Islands is estuarine, with a net-northward flow predominating during the months of our study [43]. Fish captured downstream of a salmon farm could only have arrived at that location by swimming past a salmon farm, and our results on genetic origins of the fish substantiated this. However, sockeye caught at two sites considered upstream of a salmon farm may have swum past a farm before capture because of fish movements or strong tidal currents, and the close proximity to a farm. Although we consider these occurrences infrequent, they may have contributed to the observed variability in louse infection levels observed at these sites. We placed collection sites from the north coast in a third category: no farms.

Marine Harvest Canada (MHC) is the only salmon farm company to report sea louse average abundance; raw sea louse data were not reported publicly at the time of our study. We used average *Caligus clemensi* abundance and *L. salmonis* motile abundance provided online to estimate sea louse trends on six MHC farms in the Discovery Islands during 2007–2008; sea louse data were not provided for the other 12 farms operating in the region. For periods without reported information, we calculated average abundance using the previous and subsequent values.

We performed exploratory analyses to probe for obvious spatial clusters in louse abundances for *L*. *salmonis* and *C*. *clemensi*. We used the SAS Cluster procedure with Ward's method for calculating distances between clusters; one capture site upstream of farms emerged as a clear outlier. Because such outliers can exercise undue influence on inferences based on regression-style statistical models [44], yet can also provide important insight, we singled out this site for special consideration.

We used mixed-effects modelling for formal analyses of sea lice abundances, with a random effect associated with sockeye sampling events. We performed a separate analysis for total abundance of each louse species. We used a generalized linear mixed modelling approach using SAS GLIMMIX procedure, and we specified a Poisson error distribution for lice on individual fish within a capture event. We calculated denominator degrees of freedom with a Satterthwaite [45] approximation. We included salinity, temperature, year, and position relative to salmon farms as fixed factors, as these are thought to most influence lice levels on juvenile salmon [10], [46]; position in the Discovery Islands area was set to 0 for upstream sites and 1 for downstream sites, and in the north coast to 2 for no farms. Specifically, we hypothesized that fish from locations downstream of farms would have higher louse abundance than upstream sites, that these would in turn be higher than on the north coast where there are no farms, and that high temperature and salinity would also be correlated with high lice loads (because sea louse growth in lab-based trials depends strongly on temperature and salinity [8]). This approach permitted us to test these factors simultaneously for potential influence on lice abundances. We also explored the potential contribution from an additional random factor associated with sampling sites (nested within exposure class); however, this random factor failed to contribute a significant component to the variance, and we omitted it from the final versions of the models. Finally, we ran analyses with and without the outlier site excluded. Because results were broadly similar, and due to the statistical problems of including the outlier site (mentioned above and in the Discussion section), we report findings with the outlier excluded.

We ran the complete suite of 2^4^−1 = 15 models of all subsets of the four factors on total abundance of each louse species. Because the methodology underlying GLIMMIX is based on approximations, which can generate misleading values of Akaike's Information Criterion and its variants, we used other methods to compare competing models. Specifically, we identified models for which (i) each included factor was significant, and placed further emphasis on the subset of these models for which (ii) any model containing these factors plus at least one more contained at least one factor that was not significant. That is, when we tried to add another factor, either the extra factor or a previous one already in the model became non-significant. These criteria sometimes produced more than one viable model; however, such ambiguities are to be anticipated given the correlations amongst all factors in these models (which ranged from 0.144 to 0.547). All analyses were generated using SAS/STAT software, V-9.1 (SAS Institute Inc., 2000–2004).

## Results

Genetic analyses confirmed that the majority of juvenile sockeye on the north coast were from the Skeena, Nass, and adjacent watersheds (98.3% combined), and thus they were unlikely to have been influenced by salmon farms further south before capture (Table 1; Figure 1). Conversely, all sockeye migrating through the Discovery Islands region were either from the Fraser River (85%) or nearby Johnstone and Queen Charlotte Strait rearing lakes (15%), and may have been influenced by salmon farms depending on their location.

**Table 1:** Stock proportion estimates and standard deviations for genetically identified juvenile sockeye salmon.

	North Coast 2007	Discovery Islands 2007	Discovery Islands 2008
Stock Origin	Estimate (SD)	Estimate (SD)	Estimate (SD)
Chilko Lake (Fraser River)	0.0 (0.0)	22.8 (4.7)	26.9 (3.9)
Quesnel Lake (Fraser River)	0.0 (0.1)	33.4 (5.2)	3.1 (1.9)
Shuswap Lake (Fraser River)	0.0 (0.1)	0.0 (0.2)	57.9 (4.1)
Other Fraser River	0.0 (0.2)	5.4 (2.8)	11.0 (2.7)
Washington & Oregon	0.0 (0.0)	0.0 (0.2)	0.0 (0.1)
West coast Vancouver Island	0.0 (0.1)	0.0 (0.2)	0.1 (0.4)
Johnstone & Queen Charlotte Straits	0.0 (0.1)	37.8 (4.9)	0.6 (0.6)
Queen Charlotte Strait to Skeena estuary	2.2 (0.9)	0.0 (0.5)	0.0 (0.4)
Skeena River estuary	3.1 (0.9)	0.0 (0.2)	0.0 (0.2)
Babine Lake (Skeena River)	85.0 (1.9)	0.0 (0.2)	0.0 (0.1)
Other Skeena River	7.7 (1.4)	0.0 (0.2)	0.0 (0.1)
Nass River	0.9 (1.2)	0.0 (0.2)	0.0 (0.2)
Queen Charlotte Islands	0.2 (0.4)	0.0 (0.5)	0.0 (0.3)
Southeast Alaska	0.7 (0.6)	0.6 (0.9)	0.3 (0.6)

**Figure 2:**
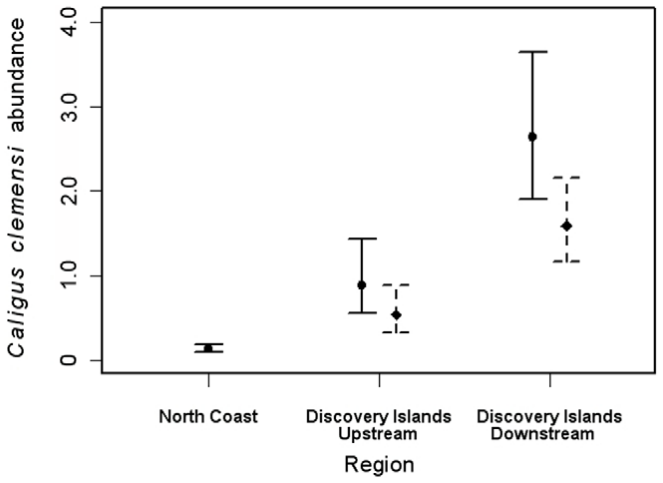
Annual estimates of *Caligus clemensi* abundance on sockeye salmon. Legend: North Coast region is without salmon farms, Discovery Islands upstream region encompasses sockeye collection sites upstream of all salmon farms given the direction of prevailing oceanic flow and migratory direction, and Discovery Islands downstream represents all collection sites downstream of farms for 2007 (solid line) and 2008 (dotted line). Estimates were obtained by back-transforming least-squares means; error bars, by back-transforming the least-squares means ±1 standard error.

Sea louse abundances on the north coast for *C*. *clemensi* and *L*. *salmonis* combined were an order of magnitude lower than in the Discovery Islands (Table 2). Within the Discovery Islands, *C*. *clemensi* was the principal louse species infecting sockeye in both years, and most abundant on fish downstream of salmon farms (Figure 2). The maximum infection intensity of *C*. *clemensi* was highest downstream of farms in 2007 (28 lice per fish) compared to upstream sites (16 lice per fish), and equal throughout the region in 2008 (9 lice per fish).

**Table 2:** Summary statistics and sea louse infection rates on juvenile sockeye.

	Position to		Total	Fork				*Caligus clemensi*				*Lepeophtheirus salmonis*			
Region	Salmon Farms	Year	Fish	Length	Mass	Salinity	Temperature	P ^a^	A b	I c	Nm d	P ^a^	A b	I c	Nm d
North Coast	No farms	2007	369	8.17 cm	5.21 g	16.97‰	9.80°C	0.09	0.17	1.97	0.97	0.01	0.01	1.00	1.00
Discovery Islands	Upstream	2007	163	7.26 cm	3.91 g	25.42‰	10.79°C	0.29	1.10	3.83	0.92	0.05	0.05	1.00	0.78
	Downstream	2007	218	7.76 cm	5.08 g	27.38‰	10.94°C	0.84	4.83	5.72	0.95	0.09	0.09	1.05	1.00
	Upstream	2008	60	8.98 cm	8.15 g	25.98‰	14.72°C	0.40	0.95	2.31	0.72	0.05	0.05	1.00	0.33
	Downstream	2008	400	10.30 cm	12.04 g	28.47‰	9.64°C	0.62	1.61	2.60	0.55	0.21	0.30	1.42	0.31
	Outlier	2008	50	9.22 cm	8.50 g	30.00‰	9.00°C	0.92	3.60	4.42	0.70	0.42	0.64	1.52	0.94

^a^Louse prevalence.

b Louse abundance.

c Louse intensity.

d Proportion of combined non-motile life stages (copepodid and chalimus I to IV).

Legend: All morphometric and abiotic values represent the mean, except sea lice infection rates.

**Figure 3:**
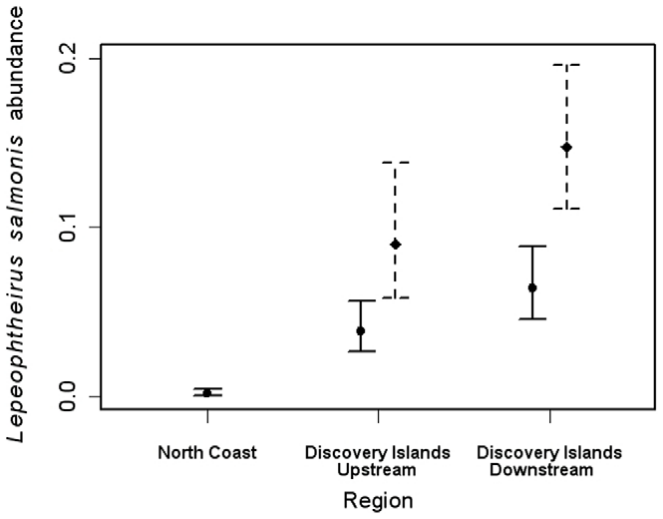
Annual estimates of *Lepeophtheirus salmonis* abundance on sockeye salmon. Legend: North Coast region is without salmon farms, Discovery Islands upstream region encompasses sockeye collection sites upstream of all salmon farms given the direction of prevailing oceanic flow and migratory direction, and Discovery Islands downstream represents all collection sites downstream of farms for 2007 (solid line) and 2008 (dotted line). Estimates were obtained by back-transforming least-squares means; error bars, by back-transforming the least-squares means ±1 standard error.

**Figure 4:**
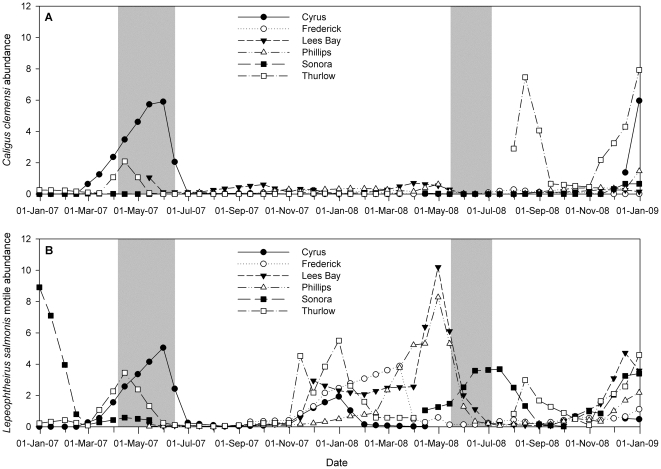
Sea louse abundance over time on Atlantic salmon on named salmon farms in the Discovery Islands. Legend: *Caligus clemensi* at top, and *Lepeophtheirus salmonis* at bottom. Period of sockeye collection during 2007 and 2008 in shaded grey.

Excluding sockeye caught at the outlier site among the Discovery Islands in 2008, which hosted the highest levels of either louse species during that year, *L*. *salmonis* was most abundant on juveniles downstream of salmon farms, and more abundant in 2008 compared to 2007 (Figure 3). In correspondence with the hypothesized contributions of salmon farms to these wild fish, MHC farms hosted more *C*. *clemensi* during the out-migration period in 2007 than 2008, and more *L*. *salmonis* in 2008 than 2007 (Figure 4).

Mixed-effects modelling showed some variation in results depending on louse species. Position relative to farms was consistently significant in all models for total abundance of *C. clemensi* in which it was included. Furthermore, whenever this factor was included, none of the others was significant; thus, the top model was clearly the one containing only this factor (*p*<0.0001). The ratio of *C. clemensi* total abundance between upstream and downstream categories was estimated by this model at 2.80 with 95% confidence intervals of 1.03 and 7.68. This ratio is significantly larger than 1 (*p* = 0.044), and *C. clemensi* abundances were significantly and substantially larger in the Discovery Islands than on the north coast (*p*≤0.0022; Figure 2).

For total abundance of *L*. *salmonis*, year was consistently significant in every model in which it appeared (*p*<0.017), although position relative to farms and salinity were also significant on their own (*p*<0.001; Table 3). Two models satisfied our selection criteria: (i) year + position relative to farms + temperature, and (ii) year + salinity + temperature; hence, the effects of position relative to farms and salinity appear confounded in these models. According to the former model, the total abundance of *L. salmonis* was significantly lower on the north coast than at each of the upstream and downstream sites in the Discovery Islands area (*p*≤0.0035), but there was no significant difference between upstream and downstream sites (*p* = 0.26). Transformed estimates derived from the least squares means for this model and their standard errors are plotted in Figure 3.

**Table 3:** Mixed effects models, and associated variance component estimates and standard errors for *Lepeophtheirus salmonis* total abundance on sockeye.

Model	Variance Component (SE)
Intercept only	1.2445 (0.3829)
Year + position to farms + temperature	0.2352 (0.1402)
Year + salinity + temperature	0.2528 (0.1550)
Salinity + temperature	0.3002 (0.1714)
Year + position to farms	0.3022 (0.1558)
Position to farms + temperature	0.3153 (0.1764)
Year + salinity	0.3697 (0.1879)
Position to farms	0.4354 (0.1969)
Salinity	0.4560 (0.2124)
Year	0.6538 (0.2691)

Legend: all factors in these models are statistically significant (*p*<0.05).

## Discussion

We have demonstrated a potential role of open net-pen salmon farms in transmission of sea lice to wild juvenile sockeye salmon. Most juvenile sockeye assessed for sea lice originated either in the Fraser or Skeena watershed, thus providing a novel comparison of sea louse infection between Canada's largest sockeye rivers. Moreover, our genetics results demonstrate a major migration corridor past farms for fish that originated in the Fraser River, a complex of populations that have been the subject of concern due to declining productivity since the early 1990s, and a collapse in 2009 followed by a substantial rebound in 2010.

Juvenile sockeye salmon in both regions were primarily infected by *C*. *clemensi*, which is consistent with juvenile pink and chum salmon in areas without salmon farms in the north Pacific [22], [49]. The predominance of *C*. *clemensi* routinely shifts to *L*. *salmonis* for pink and chum in regions with intensive salmon farming [21], [29], [47], and this was shown for those species in the Discovery Islands during the years of our study [26]. Most of the sockeye we examined among the Discovery Islands were caught in mixed schools with *L. salmonis*-infected juvenile pink and chum. Thus, the predominance of *C*. *clemensi* on sockeye upstream of farms suggests that sockeye either show higher resistance to *L. salmonis*, or heightened susceptibility to *C. clemensi*; alternatively, perhaps *C*. *clemensi* has a preference for sockeye, or *L*. *salmonis* prefers juvenile pink and chum salmon. This warrants future experimental work.

Juvenile sockeye migrating along the north coast hosted an order of magnitude fewer sea lice than those migrating through the Discovery Islands. Wild juvenile salmon in Europe and North America consistently host low levels of sea lice during their early marine migration in areas without salmon farms [22], [48], [49], though brief localized outbreaks have occurred [50], [51]. Louse parasitism of juveniles is frequently higher for sustained periods in regions with salmon farming [27], [47], [52]. Factors beyond the absence of farm salmon on the north coast may have contributed to the significantly lower lice levels on sockeye compared to the Discovery Islands. In particular, differences in lice levels may be due to our use of different sampling gear or different environmental conditions, though we did incorporate the two key conditions known to affect sea louse infection levels into our analyses: salinity and temperature. Our analyses show that the lower infection rates for *C. clemensi* on the north coast cannot be explained by salinity and temperature alone. The primary strength of our study was the comparison of infection levels before and after fish had been exposed to salmon farms within the Discovery Islands.

Parasitism of sockeye by *C. clemensi* in the Discovery Islands was higher on juveniles downstream of salmon farms than on those upstream of farms. These findings are consistent with previous research on juvenile pink and chum salmon in this region, and elsewhere in the north Pacific [26], [29]. Farm data provide further evidence that *C*. *clemensi* was abundant on farm salmon while juvenile sockeye migrated through the region, particularly during the higher infection year of 2007 [53], [54] (see our Figure 4). Although the position of sockeye relative to salmon farms was the only significant factor to explain our data, we need to consider alternative explanations. First, the spatial distribution of upstream/downstream collection sites assumes a northbound migration. Juveniles caught downstream of farms were consistently larger than upstream sockeye, which may be evidence for extended residency time (i.e., increased exposure to sea lice, which may lead to epizootics [55]). Juveniles that spent longer in the marine environment would host greater proportions of motile stage lice, as lice would have had more time to develop. However, juveniles downstream of farms primarily hosted larval stage lice, which suggests they were infected recently by a local source. Moreover, juveniles from different populations within the Fraser River are not of equal size, and they vary in their migration timing through our study region (M. Price unpublished data); thus, size may not be a simple metric for residency time and deserves further examination. Second, because *C*. *clemensi* is a generalist parasite, non-salmonids such as Pacific herring (*Clupea pallasi*) may have been a local source for lice (as has been hypothesized elsewhere [51]). We also consider this unlikely to account for *C*. *clemensi* increases on sockeye downstream of farms, as pelagic fishes would need to assume a similar spatial distribution (i.e., more fishes downstream of farms) over consecutive years, and there is no evidence for this.

Similar to *C. clemensi*, parasitism of sockeye by *L. salmonis* was higher in the Discovery Islands than the north coast, and lice levels further increased for juveniles downstream of salmon farms. Notably, the year of highest infection among the Discovery Islands was the opposite for each louse species infecting sockeye: *L*. *salmonis* was most abundant in 2008, *C*. *clemensi* was most abundant in 2007, and farm salmon in this region showed similar inter-annual trends for each species. Our mixed-effects modelling further showed that migration year best explained *L*. *salmonis* total abundance, indicating significant inter-annual variation in *L*. *salmonis* abundance on sockeye that is consistent with farm salmon. Farm salmon hosted lice well before sockeye began migrating through the region, and are the most likely source of infection.

Sockeye among the Discovery Islands were most infected with *L. salmonis* at the outlier site compared to all other sites. This site was approximately 8 km upstream from a farm salmon processing facility where large numbers of live sea lice, primarily nauplii, have recently been recorded from the effluent (A. Morton unpublished data). Tidal currents here (i.e., Discovery Passage) can transport particles this distance in a single tide-cycle [43], which suggests that the processing facility may have been a source for lice on sockeye. This also suggests that other ‘upstream’ locations may have been exposed to farm-origin lice (and may explain the significantly higher lice levels on sockeye at all upstream sites compared to the north coast), but to a lesser degree than downstream locations. Alternatively, this single location may have been home to a large congregation of resident fishes that were heavily infected with sea lice. Although we caught only sockeye during this single capture event, we have caught juvenile pink and chum salmon with relatively low lice levels at that location previously. Note that while we cannot justify including this outlier site in our formal statistical tests because it is inconsistent with the model assumptions, when we included the outlier in the analysis (the invalidity of the inferences notwithstanding), the primary conclusions remained essentially the same. Hence, this unique observation, though it does not critically impinge on the results of the study, is important in that it suggests the need for heightened attention towards the potential role of processing plants in sea lice dynamics.

Does *C*. *clemensi* pose a threat to sockeye salmon? Research to date has not examined the effects of this sea louse on wild juvenile Pacific salmonids, though significant fin damage by larval stage lice has been documented [50]. *Caligus clemensi* is smaller than *L*. *salmonis*, and is thought to cause less mechanical damage to juvenile pink and chum salmon [9], [14], [22]. Moreover, juvenile sockeye are larger and have developed scales at the time of ocean entry compared to juvenile pink and chum; thus, it is unlikely that the average number of *C*. *clemensi* observed on sockeye (2–3 lice/fish) would cause direct mortality for healthy fish. However, evidence is mounting that marine parasites, such as sea lice, can induce behavioral changes that may result in higher mortality rates for hosts [13], [56]. The transition from freshwater to marine environments is one of the most physiologically demanding phases for salmon [57], and overall marine survival appears to depend on rapid early marine growth [58]. Even low levels of parasitic infection may be harmful during this critical period. Moreover, the presence and abundance of sea lice on juvenile sockeye may be a proxy for other farm-origin pathogens. Given the high intensities of *C*. *clemensi* observed on some juveniles in this study (i.e., up to 28 lice/fish), concern is justified, and research should be undertaken to understand the extent of threat posed.

There is considerable interest in understanding the factors that affect survival of juvenile sockeye in the marine environment, and specifically whether salmon farms are contributing to declines. Sockeye productivity in many Canadian river systems has declined over the last decade, including the Skeena River; thus multiple contributing factors other than farm-origin parasites are likely responsible for reduced sockeye productivity. However, unlike most other systems, Fraser River sockeye experienced a record-low return in 2009, triggering a federal Judicial Inquiry [32]. Although the effect of sea louse parasitism on juvenile sockeye acting in isolation may arguably be small, it could be important when combined with multiple stressors [59]. Negative impacts of salmon farms on wild populations have been indicated in other parts of the world [9], [10], [60], and in juvenile pink, and coho salmon populations on the west coast of Canada [28], [61]. A recent study found no correlation between numbers of lice on farms and adult pink salmon returns in the Broughton Archipelago, which is located between our southern and northern sites [21]. This study, based on a nine-year time series, lacked full statistical comparisons of productivity in regions without salmon farms. Another recent study that included such comparisons reported significant declines in productivity of pink salmon in relation to salmon farms [62].

Our evidence suggests that salmon farms are elevating parasite levels on Fraser River sockeye during their critical early marine migration; to establish the link more definitively between farms and wild fish would require collaborative work with the salmon farm industry as has begun in Europe and the Broughton Archipelago [21], [63]. Ultimately, risks to wild salmon posed by salmon farms can be more easily mitigated than the far-reaching effects on ocean productivity of climate change and ocean acidification. Options already recommended include removal of farm salmon from migration routes of juvenile sockeye from the Fraser [64], and transitioning of salmon farms to closed-containment facilities [65]. At minimum, the Discovery Islands' migration corridor requires a co-ordinated aquaculture management plan to minimize the exposure of wild juvenile sockeye to sea lice.

## References

[1] Macdonald DW, Laurenson MK (2006). Infectious disease: inextricable linkages between human and ecosystem health.. Biol Cons.

[2] Thirgood S (2009). New perspectives on managing wildlife diseases.. J Appl Ecol.

[3] Dobson A, Foufopoulos J (2001). Emerging infectious pathogens of wildlife.. Phil Trans R Soc Lon B.

[4] Otterstatter MC, Thomson JD (2008). Does pathogen spillover from commercially reared bumble bees threaten wild pollinators?. PLoS ONE.

[5] Power AG, Mitchell CE (2004). Pathogen spillover in disease epidemics.. Am Nat.

[6] Daszak P, Cunningham AA, Hyatt AD (2000). Emerging infectious diseases of wildlife - Threats to biodiversity and human health.. Science.

[7] Jessup DA, Boyce WM, Clarke RK, Renecker LA, Hudson RJ (1991). Diseases shared by wild, exotic and domestic sheep.. Wildlife production: conservation and sustainable development.

[8] Costello MJ (2006). Ecology of sea lice parasitic on farmed and wild fish.. Trends in Para.

[9] Costello MJ (2009). How sea lice from salmon farms may cause wild salmonid declines in Europe and North America and be a threat to fishes elsewhere.. Proc R Soc Lon B.

[10] Krkosek M (2010). Sea lice and salmon in Pacific Canada: ecology and policy. Front.. Ecol Environ.

[11] Pike AW, Wadsworth SL (2000). Sea lice on salmonids: their biology and control.. Adv Para.

[12] Bjorn PA, Finstad B (1997). The physiological effects of salmon lice infection on sea trout post smolts.. Nor J Freshw Res.

[13] Krkosek M, Connors B, Mages P, Peacock S, Ford H (2010). Fish farms, parasites, and predators: implications for salmon population dynamics.. Ecol Appl. In press.

[14] Morton A, Routledge RD (2005). Mortality rates for juvenile pink salmon *Oncorhychus gorbuscha* and chum *O. keta* salmon infested with sea lice *Lepeophtheirus salmonis* in the Broughton Archipelago.. Alaska Fish Res Bull.

[15] Krkosek M, Lewis MA, Morton A, Frazer LN, Volpe JP (2006). Epizootics of wild fish induced by fish farm.. Proc Natl Acad Sci U S A.

[16] Nese L, Enger R (1993). Isolation of *Aeromonas salmonicida* from salmon lice, *Lepeophtheirus salmonis* and marine plankton.. Dis Aquat Org.

[17] Nylund A, Hovland T, Hodneland K, Nilsen F, Løvik P (1994). Mechanisms for transmission of infectious salmon anemia (ISA).. Dis Aquat Org.

[18] McCallum H, Dobson AP (1995). Detecting disease and parasite threats to endangered species and ecosystems.. Trends Ecol Evol.

[19] Orr C (2007). Estimated sea louse egg production from marine Harvest Canada farmed Atlantic salmon in the Broughton Archipelago, British Columbia, 2003-2004.. N Am J Fish Manage.

[20] Fraser NL (2009). Sea-cage aquaculture, sea lice, and declines of wild fish.. Cons Biol.

[21] Marty GD, Saksida SM, Quinn TJ (2010). Relationship of farm salmon, sea lice, and wild salmon populations.. Proc Natl Acad Sci U S A.

[22] Krkosek M, Gottesfeld A, Proctor B, Rolston D, Carr-Harris C (2007). Effects of host migration, diversity and aquaculture on sea lice threats to Pacific salmon populations.. Proc R Soc Lon B.

[23] Marine Harvest Corporate (2008). Sustainability report. Oslo, Norway.

[24] Murray AG, Peeler EJ (2005). A framework for understanding the potential for emerging diseases in aquaculture.. Prev Vet Med.

[25] Murray AG (2008). Using simple models to review the application and implications of different approaches used to simulate transmission of pathogens among aquatic animals.. Prev Vet Med.

[26] Price MHH, Morton A, Reynolds JD (2010). Evidence of farm-induced parasite infestations on wild juvenile salmon in multiple regions of coastal British Columbia, Canada.. Can J Fish Aquat Sci.

[27] Heuch PA, Bjorn PA, Finstad B, Holst JC, Asplin L (2005). A review of the Norwegian National Action Plan Against Salmon Lice on Salmonids: the effect on wild salmonids.. Aquaculture.

[28] Krkosek M, Ford JS, Morton A, Lele S, Myers RA (2007). Declining wild salmon populations in relation to parasites from farm salmon.. Science.

[29] Morton A, Routledge R, Krkosek M (2008). Sea lice infestation of wild juvenile salmon and herring associated with fish farms off the east-central coast of Vancouver Island, British Columbia.. N Am J Fish Manage.

[30] Hartt AC, Dell MB (1986). Early oceanic migrations and growth of juvenile Pacific salmon and steelhead trout.. Int N Pac Fish Com Bull.

[31] International Union for the Conservation of Nature (2008). Pacific sockeye salmon (*Oncorhynchus nerka*) added to IUCN red-list.. http://www.iucnredlist.org/apps/redlist/details/135301/0.

[32] Cohen Commission (2010). Commission of inquiry into the decline of Sockeye salmon in the Fraser River.. http://www.cohencommission.ca/en/TermsOfReference.php.

[33] Welch DW, Melnychuk MC, Rechisky ER, Porter AD, Jacobs MC (2009). Freshwater and marine migration and survival of endangered Cultus Lake sockeye salmon (*Oncorhynchus nerka*) smolts using POST, a large-scale acoustic telemetry array.. Can J Fish Aquat Sci.

[34] Peterman RM, Marmorek D, Beckman B, Bradford M, Mantua N (2010). Synthesis of evidence from a workshop on the decline of Fraser River sockeye.. http://www.psc.org/pubs/FraserSockeyeDeclineWorkshopIntro.pdf.

[35] Holst JC, McDonald A (2000). FISH_LIFT: a device for sampling live fish with trawls.. Fish Res.

[36] Kabata Z (1972). Development stages of *Caligus clemensi* (Copepoda: Caligidae).. J Res Board Can.

[37] Johnson SC, Albright LJ (1991). The developmental stages of *Lepeophtheirus salmonis* (Krflyer, 1837) (Copepoda: Caligidae).. Can J Zool.

[38] Withler RE, Le KD, Nelson RJ, Miller KM, Beacham TD (2000). Intact genetic structure and high levels of genetic diversity in bottlenecked sockeye salmon, *Oncorhynchus nerka*, populations of the Fraser River, British Columbia, Canada.. Can J Fish Aquat Sci.

[39] Beacham TE, Lapointe M, Candy JR, McIntosh B, MacConnachie C (2004). Stock identification of Fraser River sockeye salmon using microsatellites and major histocompatibility complex variation.. Trans Am Fish Soc.

[40] Pella J, Masuda M (2001). Bayesian methods for analysis of stock mixtures from genetic characters.. Fish Bull.

[41] Beacham TE, Candy JR, McIntosh B, MacConnachie C, Tabata A (2005). DNA-level variation of sockeye salmon in Southeast Alaska and the Nass and Skeena Rivers, British Columbia, with applications to stock identification.. N Am J Fish Manage.

[42] Groot C, Cooke K, Smith HD, Margolis L, Wood CC (1987). Are the migrations of juvenile and adult Fraser River sockeye salmon (*Oncorhynchus nerka*) in near-shore waters related?. Sockeye salmon (*Oncorhynchus nerka*) population biology and future management.

[43] Thomson RE (1981). Oceanography of the British Columbia coast.. Can Spec Pub Fish Aquat Sci.

[44] Kleinbaum DG, Kupper LL, Nizam A, Muller KE (2008). Applied regression analysis and other multivariable methods, fourth edition..

[45] Satterthwaite FE (1946). An approximate distribution of estimates of variance components.. Biom Bull.

[46] Brooks KM (2005). The effects of water temperature, salinity, and currents on the survival and distribution of the infective copepodid stage of sea lice (*Lepeophtheirus salmonis*) originating on Atlantic salmon farms in the Broughton Archipelago of British Columbia, Canada.. Rev Fish Sci.

[47] Krkosek M, Lewis MA, Volpe JP (2005). Transmission dynamics of parasitic sea lice from farm to wild salmon.. Proc R Soc Lon B.

[48] Gottesfeld AS, Proctor B, Rolston LD, Carr-Harris C (2009). Sea lice, *Lepeophtheirus salmonis*, transfer between wild sympatric adult and juvenile salmon on the north coast of British Columbia, Canada.. J Fish Dis.

[49] Morton A, Routledge R, Peet C, Ladwig A (2004). Sea lice (*Lepeophtheirus salmonis*) infection rates on juvenile pink (*Oncorhynchus gorbuscha*) and chum salmon (*Oncorhynchus keta*) in the nearshore marine environment of British Columbia, Canada.. Can J Fish Aquat Sci.

[50] Parker RR, Margolis L (1964). A new species of parasitic copepod, *Caligus clemensi* sp. Nov. (Caligoida: Caligidae), from pelagic fishes in the coastal waters of British Columbia.. J Fish Res Bd Can.

[51] Beamish RJ, Wade J, Pennell W, Gordon E, Jones S (2009). A large, natural infection of sea lice on juvenile Pacific salmon in the Gulf Islands area of British Columbia, Canada.. Aquaculture.

[52] Tully O, Gargan P, Poole WR, Whelan KF (1999). Spatial and temporal variation in the infestation of sea trout *Salmo trutta L*. by the caligid copepod *Lepeophtheirus salmonis* (Kroyer) in relation to sources of infection in Ireland.. Parasitology.

[53] British Columbia Ministry of Agriculture and Lands (2007). Fish health program annual report.. http://www.agf.gov.bc.ca/ahc/fish_health/fish_health2007.pdf.

[54] British Columbia Ministry of Agriculture and Lands (2008). Fish health program annual report.. http://www.agf.gov.bc.ca/ahc/fish_health/Fish_Health_Report_2008.pdf.

[55] Krkosek M, Morton A, Volpe JP, Lewis MA (2009). Sea lice and salmon population dynamics: effects of exposure time for migratory fish.. Proc R Soc Lon B.

[56] Webster SJ, Dill LM, Butterworth K (2007). The effect of sea lice infestation on the salinity preference and energetic expenditure of juvenile pink salmon (*Oncorhynchus gorbuscha*).. Can J Fish Aquat Sci.

[57] Quinn TP (2005). The behavior and ecology of Pacific salmon and trout..

[58] Beamish RJ, Mahnken C, Neville CM (2004). Evidence that reduced early marine growth is associated with lower marine survival of coho salmon.. Trans Am Fish Soc.

[59] Finstad B, Kroglund F, Strand R, Stefansson SO, Bjorn PA (2007). Salmon lice or suboptimal water quality - reasons for reduced postsmolt survival?. Aquaculture.

[60] Ford JS, Myers RA (2008). A global assessment of salmon aquaculture impacts on wild salmonids.. PLoS Biol.

[61] Connors BM, Krkosek M, Ford J, Dill LM (2010). Coho salmon productivity in relation to salmon lice from infected prey and salmon farms.. J Appl Ecol.

[62] Krkosek M, Hilborn R (2011). Sea lice (*Lepeophtheirus salmonis*) infestations and the productivity of pink salmon (*Oncorhynchus gorbuscha*) in the Broughton Archipelago, British Columbia, Canada.. Can J Fish Aquat Sci.

[63] Penston MJ, Millar CP, Davies IM (2008). Reduced *Lepeophtheirus salmonis* larval abundance in a sea loch on the west coast of Scotland between 2002 and 2006.. Dis Aquat Org.

[64] Statement from Think Tank of Scientists (2009). Adapting to change: managing Fraser sockeye in the face of declining productivity and increasing uncertainty.. http://www.sfu.ca/cstudies/science/resources/1273690566.pdf.

[65] Legislative Assembly of British Columbia (2007). Special committee on sustainable aquaculture: final report volume one.. http://www.leg.bc.ca/CMT/38thparl/session-3/aquaculture/reports/Rpt-AQUACULTURE-38-3-Volume1-2007-MAY-16.pdf.

